# Psychometric Characteristics of the German Values in Action Inventory of Strengths 120-Item Short Form

**DOI:** 10.1007/s11482-018-9696-y

**Published:** 2019-01-19

**Authors:** Stefan Höfer, Melanie Hausler, Alexandra Huber, Cornelia Strecker, Daniela Renn, Thomas Höge

**Affiliations:** 1 Department of Medical Psychology, Medical University of Innsbruck, Innsbruck, Austria; 2 Institute of Psychology, University of Innsbruck, Innsbruck, Austria

**Keywords:** Character strengths, Validity, German validation, Reliability, Positive psychology

## Abstract

The original *Values in Action Inventory of Strengths* (VIA-IS) is an international 240 item validated self-report questionnaire measuring character strengths. A validated and reliable English 120-item short form (VIA-120) is available. However, there is limited information about the psychometric properties of the German VIA-120. This article addresses this gap and reports the reliability, validity and comparability of the German VIA-120 with the German VIA-240 version. Two independent samples were recruited: a general population sample (*N* = 1073, Sample 1) and a sample consisting of medical students and physicians (*N* = 685, Sample 2). Internal consistency of the VIA-120-scales ranged from α = .58 (modesty) to α = .87 (spirituality) in Sample 1 and α = .63 (honesty) to α = .90 (spirituality) in Sample 2. Intercorrelations between the scales of the 120-item version and the original 240-Items version (Sample 1) ranged from *r* = .52 (hope) to *r* = .89 (prudence). Criterion validity with the *Satisfaction with Life Scale* (SWLS) and the *Brief Inventory of Thriving* (BIT) was demonstrated. The comparison of the factor structure between the original and the short form showed a good convergence (Tucker’s Phi .93–.99 Sample 1, .95–.98 Sample 2). Overall, the German VIA-120 was reliable, showed good convergence with the German VIA-240 and thus presents a similar level of validity for the assessment of character strengths. This study provides the first indication that the VIA 120 short form is comparable regarding the validity and reliability of the original VIA 240-item version indicating its potential to be used in large scale research studies.

## Introduction

Character strengths are postulated as a keystone for the good life, including aspects of subjective well-being, flourishing and have been defined as morally valued positive traits, reflected in thoughts, feelings and behaviors (Park et al. 2004). Based on an extensive literature review and professional consensus, the Values in Action (VIA) classification includes 24 different character strengths. These character strengths are seen as processes and mechanisms leading to six virtues, which are valued by moral philosophers and religious thinkers across time and world cultures (Peterson & Seligman, 2004). The six universal virtues include: (a) wisdom and knowledge (including the strengths of creativity, curiosity, open-mindedness, love of learning, and perspective); (b) courage (including bravery, persistence, honesty, and zest); (c) humanity (including love, kindness, and social intelligence); (d) justice (including teamwork, fairness, and leadership); (e) temperance (including forgiveness, modesty, prudence, and self-regulation); and (f) transcendence (including appreciation of beauty and excellence, gratitude, hope, humor, and spirituality) (Peterson & Seligman, 2004). Based on this classification, Peterson and Seligman (2004) chose not to measure the six virtues directly, as they were seen as too abstract. Accordingly they developed a 240-item self-report instrument that assesses the 24 character strengths in adults (10 items per strength): the VIA *Inventory of Strengths* (VIA-IS). Items are completed on a 5-point Likert scale from *very much like me (5)* to *very much unlike me (1)* and dimensionally represent each character strength. The instrument is currently available in 39 translations, including the German translation by Ruch et al. (2010) as the standard instrument for the assessment of the 24 character strengths in German-speaking samples. Satisfactory psychometric properties concerning internal consistency, test-retest reliability and validity (including self-peer agreements) of the 240-item version of the VIA-IS were shown (e.g., Ruch et al. 2010; Park et al. 2004). It has been in use over 15 years and extensive research of the VIA has been performed in relation to satisfaction with life (happiness), health and wellness, achievements or psychological problems (Niemiec 2013). However, the length of the original 240-item version is considered a disadvantage regarding time and concentration of the participant, because completion takes up to 40 min.

Currently, a few VIA short forms are available. One example is the *Character Strengths Rating Form* (CSRF), a German questionnaire including 24-items measured on a 9-point Likert scale ranging from 1 (*= not like me at all*) to 9 (*= absolutely like me*) (Ruch et al. 2014). The CSRF assesses the 24 character strengths described in the VIA classification (Peterson & Seligman, 2004) using a single rating for each strength. These ratings describe the respective character strength; Ruch et al. (2014) reported a convergence ranging from *r* = .41 to *r* = .77 with the 240 VIA-IS, which limits a valid assessment at least for some character strengths. However the main application of the CSRF is the use in large samples and it is not considered for the assessment of an individual. More short forms of the VIA-IS are for example the Spanish (Azañedo et al. 2017) or the English (Littman-Ovadia 2015) 120-item versions. The English VIA-120 consists of five items per scale. The selection of items was based on the largest corrected item-total correlations from the original 10 items per scale. It has shown to be substantially equivalent to the original English long form (VIA- 240) in reliability, validity, and factor structure. The VIA Institute on Character is currently providing this VIA-120 as the standard VIA Survey version to researchers when economy of instruments is an important criterion, replacing the original 240-item version. In particular, a short form is more economic in large studies that involve multiple measures. However, there is limited psychometric information available for the German VIA-120 version which consists of the same items as the English VIA-120 version. Hence, the aim of this report is to analyse the psychometric properties, in particular its reliability, validity and interchangeability with the German VIA-240 version by assessing internal consistency, convergent and criterion validity, and factor congruence. This was done with two different samples as part of the ongoing project “health and wellbeing of medical students and physicians” (Wellmed 2018).

## Method

### Study Design and Participants

#### Two independent samples were recruited

**Sample 1** consisted of *N* = 1109 adults who completed the German VIA-240 questionnaire, the *Satisfaction with Life Scale* (SWLS) and the *Character Strengths Rating Form* (CSRF) online as part of a paid panel survey representing the general population of Germany. Before conducting the main analyses, 36 outliers on any of the 24 scales of the VIA-240 scales were identified (critical z-value of < ±3.29, corresponding to the top/bottom 0.1% of the normal distribution) and subsequently deleted from the analysis. A control item was integrated in the VIA questionnaire to check the quality of the responses (“Please mark strongly disagree”). It was correctly answered by 100% of the final sample. The final *N* in Sample 1 consisted of 1073 data sets (55% female, mean age 45 ± 14.4 years). Most of the participants were German (95%), 98% of whom were living in Germany. The majority of the sample were in a relationship (married (39%), partnership (22%)), 31% were single and 55% reported having children. No participants had less than compulsory education, 8.6% had compulsory education, 15% of the participants completed an apprenticeship, 20% had a university-entrance diploma, while 26.9% where either currently studying or had a university degree.

**Sample 2** combined medical students and (resident) physicians working at hospitals in Austria (*N* = 705). The participants were part of a longitudinal project (e.g. Höge et al. 2019; Huber et al. 2018; Strecker et al. 2018 in this special issue) focusing on the health and well-being of medical students and practitioners. Participants completed a comprehensive set of questionnaires, including the German VIA-120, the German SWLS and the German *Brief Inventory of Thriving* (BIT). A total of 20 outliers were identified (critical z-value of < ±3.29 regarding any of the VIA-120 scales) and deleted from the sample, resulting in a final *N* of 685. The sample consisted of 63.5% females with a mean age of 26 ± 6.5 years, with 61% currently studying and 39% having a university degree. Most of the sample was from Austria (61%), Germany (18.5%) or the German speaking region of Italy (South Tyrol: 16%). More than half of them were either in a partnership (39%) or married (12%).

## Measures

### Values in Action Inventory of Strengths 240 (VIA-240)

The VIA Inventory of Strengths (VIA-IS; Peterson & Park, 2009; Peterson & Seligman, 2004) is a 240-item self-report questionnaire measuring the 24 character strengths. Respondents report to what extent these statements apply to themselves. In the present study (Sample 1), participants completed the German adaptation of the VIA-240 (Ruch et al. 2010). Items are rated on a five-point Likert scale ranging from 1 (*= very much unlike me*) to 5 (*= very much like me*). Scale scores were the arithmetic means across the items of each scale, yielding 24 scores for each participant. Higher scores indicate a higher level of the respective character strength.

### Values in Action Inventory of Strengths 120 (VIA-120)

Participants from Sample 2 completed the German 120 item version of the VIA only. The German VIA-120 is based on the English VIA-120 validation study by Littman-Ovadia (2015) (Via Institute on Character 2014). The scale ranges from 1 (= *strongly disagree)* to 5 (= *strongly agree)*. Sample items are “I have many interests” (*curiosity*) or “I feel thankful for what I have received in life” (*gratitude*).

The VIA-120 calculations for Sample 1 were carried out post-hoc based on the full VIA-240.

### Character Strengths Rating Form (CSRF)

The CSRF is a short self-report questionnaire measuring the 24 strengths (Ruch et al. 2014). Each of the items measures one of the 24 character strengths. For example, the character strength *gratitude* is measured as follows: “Grateful people are aware of and thankful for the good things that happen to them. Others describe them as being grateful, because they always take time to express thanks.” Participants can rate on a scale ranging from 1 (*= not like me at all*) to 9 (*= absolutely like me*). The German version is sufficiently valid at assessing character strengths in a very brief and economic way; however at least some character strengths show only moderate convergence with the VIA-240 (Ruch et al. 2014).

### Satisfaction with Life Scale (SWLS)

The SWLS (Diener et al. 1985) is a five-item self-report questionnaire assessing satisfaction with life in general. Each item is rated on a five-point scale ranging from 1 (= *totally disagree*) to 5 (= *totally agree*). Responses are summed to an overall score of life satisfaction. A higher score indicates a higher life satisfaction. The SWLS has been demonstrated to possess adequate psychometric properties in several studies (see Pavot & Diener, 2008). As character strengths are in general linked with satisfaction with life (Peterson et al. 2007), this scale was chosen to test the criterion validity of the VIA – 120. In the current study, we applied the German version of the SWLS (Glaesmer, Grande, Braehler, & Roth 2011), which showed a good internal consistency in Sample 1 (α = .90) and Sample 2 (α = .82).

### Brief Inventory of Thriving (BIT)

The BIT is a 10-item screening instrument that assesses self-reported comprehensive well-being (including aspects of subjective and psychological well-being) and is the short form of *Comprehensive Inventory of Thriving* (CIT) (Su et al. 2014). The scales range from 1 (= *strongly disagree*) to 5 (= *strongly agree*). Sample items are “My life is going well”, “I am achieving most of my goals” and “My life has a clear sense of purpose”. Pleasure, flow, thriving among other positive experiences are theoretically enabled by character strengths (Peterson et al. 2007). Therefore the BIT was chosen to test the criterion validity of the VIA short form. The German version is valid and reliable (Hausler et al. 2017a). In the present study the BIT has an acceptable internal consistency with α = .81 in Sample 2.

### Data Analyses

First of all, we described the results of the character strengths based on the different instruments. To evaluate reliability, alpha coefficients of the original VIA-240 (Sample 1) were compared with the VIA-120 in the same sample and with those that were administered independently (Sample 2). Mean inter-item correlations per scales were calculated to assess the overlap between the items independent of the number of items in each scale. To evaluate convergent and criterion validity, firstly, correlations between the SWLS and the CSRF with the VIA-240 scales were compared with those with the VIA-120 scales (Sample 1). Secondly, the VIA-120 scales, were correlated adjusted for item overlap (Cureton 1966) with their respective VIA-240 in the same sample (Sample 1) to address convergence. This correlation was calculated using the function scoreOverlap of the r-package psych 1.8.4 (Revelle (2018) in R (R Core Team 2018). Thirdly, the correlations between the SWLS, the BIT and the VIA-120 were analyzed for Sample 2 as well. Based on previous findings (Hausler et al. 2017a) we expected in particular high correlations between SWLS and character strengths Curiosity, Gratitude, Hope, Humor, Love, Spirituality and Zest. Finally, factor analysis results of the VIA-240 were compared with those for the VIA-120 (both samples). To estimate the level of congruence between the two versions Tucker’s phi was calculated (Lorenzo-Seva and ten Berge 2006). For the interpretation of correlation coefficients Cohen’s effect size criteria were applied (correlations of < .10 = none, .10–.29 = weak, .30–.49 = moderate, ≥ 50 = high, Cohen 1992).

## Results

### Descriptives and Reliability of the Instruments (Sample 1 and Sample 2)

Means and standard deviations for both samples are reported in Table 1. The means of the 24 character strengths, on a potential range from 1 to 5, ranged from 2.45 (spirituality, VIA-120, Sample 2) to 4.25 (honesty, VIA-120, Sample 2).

Standard deviations ranged from 0.45 (honesty, VIA-120, Sample 2) to 1.02 (spirituality, VIA-120, Sample 2). Skewness and kurtosis of the VIA-240 and the VIA-120 (Sample 1 and Sample 2) were in an acceptable range (±1). The three character strengths with the highest means were the same in both samples regardless of the VIA-questionnaire versions: honesty, kindness and fairness. The character strength spirituality had the lowest mean score, independent of the sample or instrument used (Table 1). The means of the CSRF scales as well as the differences regarding the level of character strengths based on the VIA-120 between the two samples can be found in Table 1.

The internal consistencies of the 24 VIA-120 scales ranged from α = .58 (modesty, Sample 1) to α = .90 (spirituality, Sample 2). The alphas of the VIA-240 scales (Sample 1) ranged from α = .75 (modesty) to α = .91 (spirituality). The average alpha of the VIA-240 (α = .82) was slightly higher compared to the VIA-120 (α = .76, α = .72), which can be expected due to the higher number of items per scale (10 vs. 5). In 8 out of 24 scales Cronbach’s alpha of the VIA 120 (Sample 1 and 2) was more than 0.10 points lower than the VIA-240 version, with a maximum divergence of .18 points for the character strength teamwork. Average inter-item correlations per scale for all versions ranged from 0.23–0.47 (VIA 240), 0.22–0.57 (VIA 120, Sample 1) and 0.26–0.63 (VIA 120, Sample 2). Acceptable values range from 0.15 to 0.50 (Clark and Watson 1995), with less than 0.15 indicating poor inter-correlation and greater than 0.50 indicating content repetition. Overall the average inter-item correlation showed good overlap, however, the scale spirituality of the VIA 120 reached in both samples critical values (0.57; 0.63 respectively) indicating that the meaning of the items could be considered repetitive (Table 1). The comparison of the VIA 240 and VIA 120 scale scores showed nevertheless a high overlap (Table 1).

Sample 1 had significantly lower SWLS scores (23.14 ± 6.37) than Sample 2 (26.74 ± 4.96) (*p* < .001). The BIT had a mean of 4.06 (±.48).

The correlation among the scales for the VIA 240 ranged from 0.22 (Spirituality x Enthusiasm) to 0.78 (Hope x Teamwork), for the VIA 120 (Sample 1) from 0.10 (Modesty x Curiosity) to 0.77 (Enthusiasm x Humor) and for the VIA 120 (Sample 2) from 0.08 (Courage x Modesty) to 0.49 (Open minded x Love of learning).

### Validity: Associations of the VIA with Measures of Well-Being and Strengths

#### Convergent Validity (Sample 1)

Correlations adjusted for item overlap between the scales of the VIA-240 and the respective scales of the VIA-120 (Sample 1) showed high, effect sizes ranging from *r* = .52 (hope) to *r* = .89 (prudence), with a median correlation of *r = .77* (Table 1). The correlation between the rank orders of the strengths obtained in the VIA-240 and the VIA-120 Sample 1 was .86 (Sample 1).

The scales of the CSRF were correlated with the respective VIA-240 and VIA-120 scores in Sample 1. Correlations were all highly significant and ranged from *r* = .44 (curiosity, VIA-120 Sample 1) to *r* = .74 (spirituality, VIA-240, 120 both Sample 1), with only two character strengths showing moderate correlations: curiosity (*r = .44*) and prudence (*r = .46*) with the CSRF. The correlation between the rank orders of the strengths obtained in the CSRF and VIA-120 Sample 1 was .76.

#### Criterion Validity (Sample 1 and Sample 2)

Regarding the criterion validity, the VIA-240 scales were correlated with the SWLS and the CSRF. The VIA-120 scales obtained from the same sample (Sample 1) and the independently administered VIA-120 (Sample 2) were both correlated with the SWLS. Moreover, the well-being measure BIT was correlated with the VIA-120 scale (Sample 2).

Correlations with the SWLS ranged from *r* = .14 (modesty, VIA 240) to *r* = .56 (hope, VIA 240), from *r* = .12 (modesty, VIA-120, sample) to *r* = .58 (hope, VIA-120, Sample 1) and from *r* = .05 (modesty) to *r* = .52 (hope, VIA-120 Sample 2, Table 2). Correlation coefficients showed equally strong effect sizes for all versions of the VIA for the character strengths hope and zest, equally moderate for Curiosity, Gratitude and Love and equally weak for appreciation of beauty and excellence, fairness, honesty, learning, prudence and social intelligence. Inconsistent correlations were found between the VIA-240 and the VIA-120 in both samples for forgiveness, humor, perseverance, perspective, self-regulation, and teamwork with all at least moderate correlations between the VIA-240 and the SWLS and only weak correlations between both VIA-120 samples and the SWLS. Further, the VIA-120 scales in the independently administered Sample 2 showed lower correlations with the SWLS. All correlations were significant except for modesty, courage, creativity, and spirituality.

The correlations of the two VIA versions in the two samples with the SWLS were tested for significant differences. The correlations of the VIA 240 and VIA 120 (both Sample 1) with the SWLS were not significantly different from each other; however 14 correlations of the VIA120 (Sample 2) with the SWLS were significantly lower than the respective VIA 240 correlations. Only 5 correlations of the VIA 120 (Sample 2) with the SWLS were significantly lower than the respective VIA 120 correlations (Sample 1).

The hypothesized highest correlations between the character strengths curiosity, gratitude, Hope, love and zest (any VIA questionnaire version, both samples) and SWLS could be found in the data (Table 2; all r ≧30); Also Humor (any VIA questionnaire version, both samples) and SWLS were significantly correlated with SWLS however only at r .20. Spirituality was only related for the VIA-240 and VIA-120 (Sample 1) with SWLS *r* = .27, *r*=. 22 respectively and not on VIA-120 (Sample 2, *r* = .06).

A similar pattern could be found for the correlations of the BIT with the VIA-120 (Sample 2). Curiosity, Gratitude, Hope, Humor, Love and Zest were significantly correlated with the BIT having a moderate to high effect size. Spirituality was significantly correlated with the BIT, however only with a weak effect size.

### Factor Structure of the German 120-Item Version of the VIA-IS (Sample 1 and Sample 2)

Factor structures (principal axis factoring with promax rotation, power = 4) of the scales of VIA-120 (both samples) were compared with the factor structure of the VIA-240 (Sample 1, Table 3). The previously established five-component solution could be replicated with both versions (e.g., Littman-Ovadia & Lavy, 2012; Ruch et al. 2010). The variance accounted for 64.38% and 57.82% in the data for the VIA-120 and 72.69% for the VIA-240. In line with previous studies (e.g., Ruch et al. 2010; Littman-Ovadia 2015; Azañedo et al. 2017) the five factors identified were labelled as follows: (1) *emotional* character strengths, (2) *interpersonal* character strengths, (3) character strengths of *restraint*, (4) *intellectual* character strengths, and (5) *theological* character strengths. We found that seven VIA-240 scales and seven VIA-120 (Sample 1) scales demonstrated double loadings (difference ≤ .10 between scales’ loadings, criterion according to Ruch et al. 2010) and that two character strengths (hope and open-mindedness) of the VIA-120 did not load on the same factors as in the VIA-240 (*theological* vs. *emotional* and *restraint* vs. *intellectual* character strengths). Further the character strength love in both versions did not load as in previous publications on the factor *emotional* character strengths (Littman-Ovadia 2015; Ruch et al. 2010) but on the factors *interpersonal* (VIA-240) and *theological* strengths (VIA-120). Tucker’s Phi indicated for the VIA-120 an acceptable level of congruence with the VIA – 240 ranging from .93 (intellectual character strengths) to .99 (emotional, interpersonal, restraint and theological character strengths) for Sample 1 and .95 (emotional, interpersonal and intellectual character strengths), .97 (theological character strengths) and .98 (restraint character strengths) for Sample 2.

Our results indicated a sufficient convergence between the factor structures of the original and the short form (Table 3).

## Conclusion

The present analysis explored the psychometric properties of the German VIA-120 version in two independent samples. Our results were mostly consistent with other reports regarding the psychometric properties of the English (Littman-Ovadia 2015) or the Spanish (Azañedo et al. 2017) short forms. Some of the scales of the VIA-120 fell below acceptable thresholds for reliability, in particular modesty and self-regulation (both samples). Also the validity of the independent administered VIA - 120 (Sample 2) with the respective SWLS was less evident than compared to the full VIA – 240. However, based on the length of the instrument, the VIA-120 can be considered a more economical way to measure the character strengths as proposed by Peterson and Seligman (2004) and can be recommended accordingly for the use primarily in large research projects still allowing the assessment of individual’s character strengths. Using the same items in the German VIA-120 as in the original English VIA-120 (Littman-Ovadia 2015) allows for international comparison studies (e.g. cultural differences). As further alternative for large scale research projects when there are certain constraints regarding the study design (i.e. length of instruments being used) the CSRF based on the presented results can also be recommended. Nevertheless, the full VIA-240 remains the gold standard when it comes to assessing an individual’s character strengths, as it covers the full breadth of behaviors related to the character strengths.

There are several limitations and starting points for future research. First of all, Sample 2 is very specific. Medical students and physicians may differ regarding their profile of character strengths compared to the general population. This was indeed found in our data (Table 1). The means of the character strengths (VIA-120) were compared regarding statistical significance between Sample 1 (general population) and Sample 2 (medical students and physicians). Sample 2 had significantly higher mean values in 13 out of 24 character strengths compared to the general population, while only 3 character strengths values were significantly higher in the general population. No difference was found between the samples for 8 character strengths values. These differences in samples may also be a reason for the different results regarding the correlations of the VIA-120 and the satisfaction with life scale.

Secondly, further examinations of the validity are needed, for example, by comparing self-ratings with peer ratings, using the scales in order to predict non-self-report behaviors and to analyze the stability of the 24 scales over time. Recently, McGrath (2017) published some revised questionnaire versions of the original VIA-240. The revisions included several improvements, e.g., by selecting the most appropriate items and shortening their number, including also negatively keyed items, or considering the wording of the items. A revision may also be taken into consideration for the German versions, in order to further improve validity and reliability of the measures and ensure international comparability. Another point to further examine is the factor structure. In the present study, we were guided by the analyses of Ruch et al. (2010), McGrath (2014), Littman-Ovadia (2015), and Azañedo et al. (2017), who found the VIA-IS 240 and the VIA-120 scales are best represented by a five-factor model. McGrath (2017) also summarized, that this model seems to fit best, but he also mentioned another possible factor model including three factors. The three-factor model was replicable across several studies and the factors were called Caring, Inquisitiveness, and Self-Control (McGrath, 2015). The five-factor model showed an overlap with the six-virtue model stated by Peterson and Seligman, but also the three-factor model mirrored several approaches regarding the concept of virtue (McGrath 2017). However, the second order factors of character strengths are not virtues and therefore cannot be seen as an indicator for providing empirical evidence for the VIA classification (Ruch and Proyer 2015).

Thirdly, the reduction of items by 50% per scale limits the assessment of the breadth of the content of the character strengths by using a mostly homogenous set of items. This is a general critique for any short form, however it would be worthwhile to address this question empirically by using different items sets of the VIA 240 to gain empirical evidence of this dilemma.

Finally, as parts of our analyses are utilized post-hoc calculations based on the full VIA-240 the convergence estimates are probably upper-bound estimates (Smith et al. 2000). Independent administrations of the VIA-120 (as carried out with Sample 2) potentially provide a better basis for comparing the VIA-120 short form with the VIA-240 long form.

As a general remark, it shall be noted that the samples differed from each other (age, gender, education, level of character strengths, etc.), and it is common sense that psychometric properties are not the sole function of an instrument but also sample dependent. The student sample might have been more homogeneous in relation to character strengths in general. Future analysis based on item-response theory might enhance the methodology of assessment of character strengths.

## Figures and Tables

**Table 1:** Character strength measures

	Sample 1											Sample 2				Sample 1 vs 2
Scales	CSRF		VIA - 240				VIA - 120				VIA - 240 x VIA - 120 ^§^	VIA - 120				VIA - 120^#^
	M (SD)	R	M (SD)	R	alpha	AIIR	M (SD)	R	alpha	AIIR	*r*	M (SD)	R	alpha	AIIR	*p* value
Beauty	6.53 (1.66)	12	3.48 (.55)	20	0.79	0.28	3.61 (.61)	12	0.73	0.36	.74**	3.54 (.69)	17	0.72	0.35	.033
Courage	5.96 (1.63)	23	3.62 (.49)	12	0.78	0.26	3.64 (.56)	10	0.71	0.33	.71**	3.53 (.63)	18	0.72	0.34	<.001
Creativity	6.34 (1.82)	20	3.46 (.61)	22	0.88	0.43	3.47 (.66)	19	0.85	0.54	.85**	3.41 (.69)	21	0.84	0.51	.106
Curiosity	7.12 (1.44)	3	3.78 (.51)	4	0.81	0.31	3.66 (.58)	9	0.76	0.4	.77**	3.84 (.57)	8	0.72	0.35	<.001
Fairness	7.08 (1.37)	5	3.90 (.48)	1	0.82	0.31	3.90 (.56)	3	0.77	0.4	.74**	4.10 (.55)	3	0.73	0:35	<.001
Forgiveness	6.35 (1.59)	19	3.48 (.53)	21	0.81	0.3	3.51 (.62)	17	0.73	0:35	.77**	3.53 (.63)	19	0.67	0.29	.414
Gratitude	6.91 (1.54)	9	3.71 (.55)	8	0.84	0:36	3.54 (.67)	15	0.79	0:43	.78**	3.66 (.62)	13	0.75	0.39	<.001
Honesty	7.30 (1.42)	2	3.87 (.46)	2	0.77	0.27	4.17 (.47)	1	0.7	0.32	.72**	4.25 (.45)	1	0.63	0.26	<.001
Hope	6.53 (1.73)	13	3.59 (.57)	15	0.84	0.35	3.64 (.66)	11	0.77	0.41	.52**	3.80 (.63)	10	0.67	0. 3	<.001
Humor	7.01 (1.59)	7	3.78 (.54)	5	0:87	0.4	3.77 (.63)	8	0.81	0.46	.84**	3.82 (.68)	9	0.82	0.47	.203
Kindness	7.38 (1.33)	1	3.85 (.48)	3	0.77	0.28	4.03 (.53)	2	0.78	0.42	.77**	4.21 (.50)	2	0.74	0.36	<.001
Leadership	6.20 (1.82)	21	3.60 (.51)	13	0.82	0.32	3.56 (.56)	14	0.72	0.34	.78**	3.71 (.53)	12	0.65	0.29	<.001
Learning	6.63 (1.66)	11	3.53 (.63)	17	0.84	0.37	3.46 (.77)	20	0.78	0.41	.81**	3.55 (.72)	16	0.74	0.36	.019
Love	6.99 (1.71)	8	3.73 (.55)	7	0.8	0.29	3.85 (.66)	5	0.77	0.41	.77**	4.05 (.62)	4	0.7	0.33	<.001
Modesty	6.48 (1.64)	14	3.49 (.49)	18	0.75	0.23	3.39 (.54)	22	0.58	0.22	.68**	3.33 (.65)	22	0.67	0.29	.033
Open-minded	7.05 (1.33)	6	3.78 (.49)	6	0.85	0.37	3.84 (.57)	6	0.79	0.39	.75**	4.04 (.55)	5	0.72	0.34	<.001
Perseverance	6.69 (1.63)	10	3.67 (.55)	10	0.86	0.39	3.87 (.58)	4	0.8	0.44	.80**	3.91 (.62)	7	0.76	0.4	.111
Perspective	6.47 (1.53)	15	3.57 (.48)	16	0.81	0.28	3.39 (.58)	21	0.75	0.38	.76**	3.53 (.59)	20	0.7	0.31	<.001
Prudence	6.39 (1.71)	17	3.49 (.53)	19	0.78	0.43	3.51 (.62)	16	0.76	0.43	.89**	3.55 (.63)	15	0:72	0.4	.185
Self-regulation	6.00 (1.84)	22	3.37 (.55)	23	0.77	0.26	3.18 (.70)	23	0.69	0.31	.70**	3.23 (.72)	23	0.67	0.29	.091
Social intelligence	7.12 (1.46)	4	3.68 (.47)	9	0.79	0.29	3.78 (.54)	7	0.76	0.41	.76**	3.92 (.52)	6	0.69	0.31	<.001
Spirituality	4.07 (2.6)	24	2.69 (.87)	24	0.91	0.48	2.47 (.99)	24	0.87	0.57	.79**	2.45 (1.02)	24	0.9	0.63	.769
Teamwork	6.46 (1.73)	16	3.64 (.50)	11	0.81	0.31	3.58 (.57)	13	0.76	0.4	.75**	3.73 (.51)	11	0.63	0.27	<.001
Zest	6.39 (1.67)	18	3.60 (.55)	14	0.83	0.33	3.48 (.64)	18	0.8	0.44	.80**	3.61 (.65)	14	0.76	0.39	<.001

Sample 1: N = 1073; Sample 2: N = 685; VIA - 240: Values in Action Inventory of Strengths original 240 Item; VIA - 120: Values in Action Inventory of Strengths 120 Item; CSRF = Character Strengths Rating Form; R = rank of means (1 indicates numerically highest mean and 24 indicates the numerically lowest mean of the scores); AIIR = average inter item correlation; Beauty = Appreciation of Beauty and Excellence

§ adjusted for item overlap

# *p*-values calculated with the independent sample t-test

** statistical significance level of *p* < .01

**Table 2:** Reliability and validity statistics

Scales	Validity					

	SWLS			CSRF		BIT
	VIA-240	VIA-120_1	VIA-120_2	Via- 240	VIA-120_1	VIA-120_2
Beauty	.25**	.19**	.10*	.59**	.53**	.18**
Courage	.28**	.21**	.06	.54**	.51**	.20**
Creativity	.20**	.20**	.08	.67**	.62**	.17**
Curiosity	.44**	.43**	.35**	.47**	.44**	.46**
Fairness	.22**	.18**	.18**	.57**	.55**	.29**
Forgiveness	.31**	.25**	.14**	.62**	.57**	.22**
Gratitude	.40**	.41**	.35**	.67**	.64**	.41**
Honesty	.22**	.24**	.20**	.56**	.52**	.32**
Hope	.56**	.58**	.52**	.63**	.65**	.67**
Humor	.33**	.28**	.23**	.68**	.70**	.33**
Kindness	.25**	.22**	.19**	.58**	.58**	.27**
Leadership	.27**	.25**	.18**	.59**	.57**	.26**
Learning	.28**	.24**	.11**	.61**	.52**	.18**
Love	.43**	.44**	.38**	.61**	.60**	.41**
Modesty	.14**	.12**	.05	.55**	.53**	.08*
Open-minded	.22**	.19**	.11*	.58**	.55**	.13**
Perseverance	.36**	.29**	.22**	.63**	.59**	.34**
Perspective	.31**	.28**	.19**	.56**	.54**	.26**
Prudence	.27**	.20**	.15**	.48**	.46**	.19**
Self-regulation	.31**	.25**	.15**	.54**	.48**	.21**
Social intelligence	.28**	.23**	.20**	.56**	.57**	.30**
Spirituality	.27**	.22**	.06	.74**	.74**	.13**
Teamwork	.30**	.26**	.21**	.58**	.60**	.28**
Zest	.51**	.55**	.50**	.63**	.61**	.64**

VIA-240; VIA-120_1, N = 1073; VIA-120_2, Sample 2: N = 685; SWLS = Satisfaction with Life Scale; CSRF = Character Strengths Rating Form; BIT = Brief Inventory of Thriving; Beauty = Appreciation of Beauty and Excellence; Pearson’s correlation coefficients (two tailed): correlations of < .10 = none, .10–.29 = weak, .30–.49 = moderate, ≥ 50 = high; *=statistical significance level of *p*=< .05; **= statistical significance level of *p* < .01

**Table 3:** Factor analyses* structure matrix results for the German original VIA-240 and the VIA-120 (Sample 1 and Sample 2)

	VIA-240 factors					VIA-120_1					VIA-120_2				
	1	2	3	4	5	1	2	3	4	5	1	2	3	4	5
Beauty	.5	.58	.33	.66	**.67**	.48	.49	.31	.18	**.64**	.31	.33	.19	.28	**.5**
Courage	**.84**	.52	.39	.51	.34	**.74**	.3	.33	.37	.27	.4	.16	.31	**.53**	.17
Creativity	.7	.39	.2	**.77**	.34	**.69**	.26	.27	**.69**	.35	.36	.16	.2	**.71**	.25
Curiosity	.64	.51	.37	**.79**	.52	**.66**	.42	.33	**.65**	.61	.4	.25	.32	**.75**	.55
Fairness	.5	**.84**	.55	.46	.32	.45	**.84**	.36	.17	.36	.4	**.82**	.39	.29	.35
Forgiveness	.43	**.71**	.51	.3	.52	.27	**.67**	.41	.08	.49	.18	**.5**	.24	.19	.42
Gratitude	.58	.72	.43	.46	**.82**	.5	.56	.4	.19	**.87**	.37	.45	.31	.32	**.78**
Honesty	**.68**	.66	.61	.3	.25	**.71**	.57	.51	.02	.36	.42	.52	**.61**	.2	.31
Hope	**.79**	.56	.38	.5	.68	.66	.43	.35	.33	**.76**	.44	.23	.28	.45	**.72**
Humor	**.7**	.66	.11	.38	.52	**.71**	.45	.06	.19	.52	**.55**	.23	.06	.43	.4
Kindness	.61	**.85**	.35	.38	.51	.6	**.77**	.26	.04	.56	**.58**	.58	.27	.24	.45
Leadership	.66	**.76**	.41	.57	.33	.64	**.67**	.41	.4	.38	**.52**	.64	.46	.43	.33
Learning	.49	.4	.31	**.87**	.37	.35	.28	.29	**.77**	.35	.15	.25	.31	**.55**	.26
Love	.67	**.75**	.26	.33	.63	.6	.52	.22	.09	**.67**	.39	.29	.24	.18	**.45**
Modesty	.23	.51	**.75**	.1	.33	.18	.52	**.66**	−.15	.38	−.01	**.53**	.43	.01	.3
Open-minded	.69	.46	.67	**.7**	.19	.61	.36	**.69**	.46	.2	.28	.23	**.68**	.38	.1
Perseverance	**.8**	.5	.61	.47	.35	**.66**	.46	.62	.2	.4	.23	.33	**.62**	.27	.31
Perspective	**.82**	.47	.53	.58	.28	**.7**	.28	.49	.41	.32	.44	.1	**.51**	.46	.22
Prudence	.58	.45	**.86**	.47	.29	.5	.36	**.86**	.28	.29	.25	.39	**.8**	.18	.22
Self-regulation	.61	.4	**.75**	.51	.35	.32	.25	**.7**	.34	.41	.1	.3	**.51**	.26	.34
Social intelligence	**.82**	.64	.4	.52	.35	**.74**	.62	.33	.22	.48	**.69**	.37	.36	.33	.34
Spirituality	.37	.35	.23	.34	**.82**	.19	.24	.2	.25	**.71**	.06	.18	.08	.06	**.4**
Teamwork	.54	**.86**	.36	.38	.43	.46	**.8**	.25	.17	.41	.38	**.71**	.34	.26	.38
Zest	**.83**	.61	.36	.53	.63	**.72**	.43	.29	.43	.73	.51	.23	.25	.64	**.71**

VIA-240 = Values in Action Inventory of Strengths original 240 Item version (N = 1073;); VIA_120_1: Values in Action Inventory of Strengths 120 Item version, (N = 1073) 1; VIA_120_2: Values in Action Inventory of Strengths 120 Item version, (N = 685) 1 emotional 2 Interpersonal, 3 restraint, 4 intellectual strengths, 5 theological Beauty = Appreciation of Beauty and Excellence; * = Promax rotated 5 factor-solution (principal axis factoring) with promax rotation, power = 4 Bold: highest factor loadings per factor
